# Two novel compound heterozygous mutations in NGLY1as a cause of congenital disorder of deglycosylation: a case presentation

**DOI:** 10.1186/s12881-020-01067-1

**Published:** 2020-06-23

**Authors:** Haixia Ge, Qingbin Wu, Huigang Lu, Yong Huang, Tingting Zhou, Danlin Tan, ZhongqinJin

**Affiliations:** Department of Gastroenterology, Children’s Hospital of Soochow University, Suzhou, Jiangsu China

**Keywords:** NGLY1, Congenital disorder of deglycosylation, Elevated transaminase developmental delay, Seizure, Constipation

## Abstract

**Background:**

NGLY1-related congenital disorder of deglycosylation (NGLY1-CDDG) is a multisystemic neurodevelopmental disorder in which affected individuals show developmental delay, epilepsy, intellectual disability, abnormal liver function, and poor growth. This study presents a 10-month-old female infant with elevated liver transaminases, developmental delay, epilepsy (subclinical seizures), and constipation who possesses two compound heterozygous mutations in *NGLY1*.

**Case presentation:**

The proband was admitted to the Department of Gastroenterology, Children’s Hospital of Soochow University, with elevated liver transaminases. She had a history of intrauterine growth retardation and exhibited elevated transaminases, global developmental delay, seizures and light constipation during early infancy. Whole-exome sequencing (WES) and Sanger sequencing revealed two compound heterozygous mutations in *NGLY1* that had been inherited in an autosomal recessive manner from her parents. One was a termination mutation, c.1168C > T (p.R390*), and the other was a missense mutation, c.1156G > T (p.D386Y). NGLY1-CDDG is a rare disorder, with a few dozen cases. The two mutations of this proband has not been previously identified.

**Conclusions:**

This study investigated a Chinese proband with NGLY1-CDDG born from healthy parents who was studied using WES and Sanger sequencing to identify the causative mutations. We identified two novel compound heterozygous mutations in *NGLY1*, c.1168C > T (p.R390*)/c.1156G > T (p.D386Y), which are probably causative of disease.

## Background

NGLY1-CDDG is a multisystemic neurodevelopmental disorder (Online Mendelian Inheritance in Man: 615273). It is an autosomal recessive disorder with characteristic of mild to profound developmental delay and intellectual disability, elevated liver transaminases, hypoalacrima, and a complex hyperkinetic movement disorder that can include choreiform, athetoid, dystonic, myoclonic, action tremor, and dysmetric movements [1–4].

NGLY1-CDDG was first described by Need AC et al. They used whole-exome sequencing to study undiagnosed genetic diseases, and found two loss-of-function mutations in a patient who may have a congenital disorder of glycosylation [5]. NGLY1-CDDG is caused by mutations in *NGLY1*, which encodes the enzyme N-glycanase 1, which is involved in the deglycosylation of glycoproteins, an essential step in the endoplasmic reticulum-associated degradation (ERAD) pathway [6]. NGLY1-CDDG is a rare disease, and only a few dozen cases have been described. One case was from China [7]. Here, we describe a Chinese infant with elevated liver transaminases, developmental delay, epilepsy (subclinical seizures) and light constipation who possesses two novel compound heterozygous mutations in *NGLY1*: a missense mutation and a termination mutation.

## Case report

In 2017, a 10-month-old female infant was admitted to the Department of Gastroenterology, Children’s Hospital of Soochow University, because of a history of elevated liver transaminases for more than 3 months. She was born at 30 weeks of gestation, her birth weight was 2.35 kg, and she had a history of intrauterine growth retardation. She had shown global developmental delay since birth. She defecated once every 3–4 days and was crying and restless during defecation. Her parents were physically healthy and were unrelated. She had a brother who died of “convulsion” at the age of 10 months, and there was no family history of inherited diseases.

**Fig. 1:**
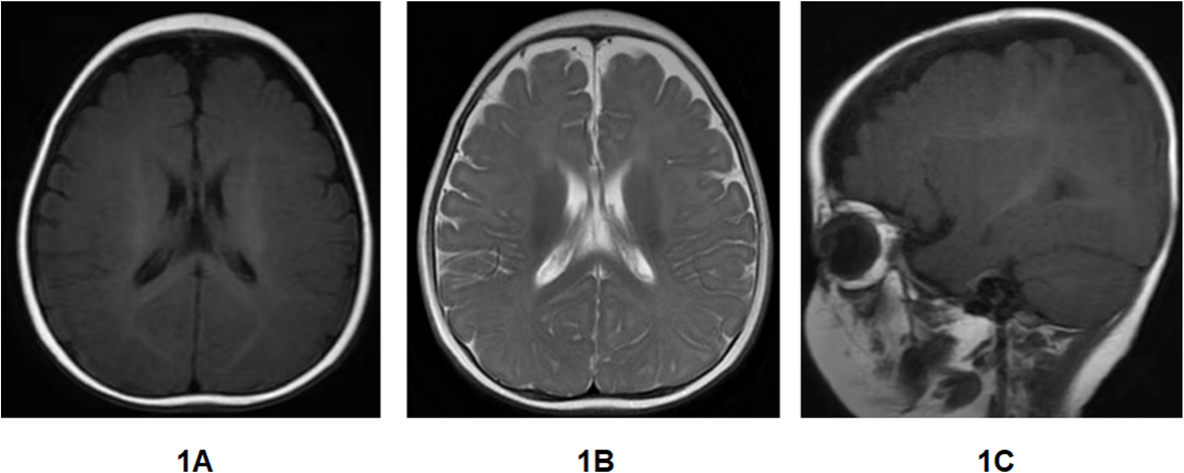
Magnetic resonance imaging of the proband’s brain. 1A Transverse position (T1WI). An increased distance can be seen between the left frontal lobe and the inner plate of the adjacent skull. 1B Transverse position (T2WI). The increased extracerebral space in the left forehead is filled with cerebrospinal fluid, and the local sulcus is widened. 1C Sagittal position (T1WI). Widening of the extracerebral space is seen in the prefrontal lobe

Physical examination was normal except for slightly high ankle tension. Her liver biochemical profile revealed elevated levels of alanine transaminase (147 U/L; normal range, 5–40 U/L) and aspartate transaminase (112 U/L; normal range, 8–40 U/L). Blood tests revealed mildly elevated levels of lactate (4 mmol/L; normal range, 0.5–2.5 mmol/L) and normal levels of IgG, IgA, IgM, and IgE immunoglobulins. Lymphocyte subset analysis was normal, as was blood coagulation function, thyroid function, blood tandem mass spectrometry, and levels of trace elements, ammonia, alpha foetal protein, and urine reducing substances. Pathogen testing was positive for cytomegalovirus IgM, and PCR for cytomegalovirus DNA in peripheral blood revealed the presence of 2.54 × 10^3^ copies/ml; other pathogens such as Epstein-Barr virus and hepatitis A, B, C, and E were all negative. Ambulatory electroencephalography (EEG) monitoring suggested epilepsy in the form of subclinical seizures. Magnetic resonance imaging of the brain demonstrated increased extracerebral space (Fig. 1). After the patient admission into hospital, she was treated with rehabilitation training and oral compound glycyrrhizin tablets (2.5 mg/kg per day) for 10 days. Liver transaminase levels were slightly reduced compared to the first presentation.

## Genetic testing and results

With the consent of the patient’s parents, whole exome sequencing (WES) was carried out to screen the mutational sites. Peripheral blood samples from the proband and his parents were collected and sent to Running Gene Inc. (Beijing, China). Following the manufacturer’s instructions, DNA samples were extracted using DNA Isolation Kits (Bioteke, Beijing, China; AU1802) and prepared as Illumina sequencing libraries using KAPA Library Preparation Kit (Kapa Biosystems, Beijing, China; KR0453). The libraries were estimated with Qubit dsDNA HS Assay kits (Invitrogen, CA, USA; Q32851). Hybridization of the pooled libraries and removal of non-hybridized DNA fragments were carried out according to the Agilent SureSelectXT2 Target Enrichment System (Agilent, CA, USA). Captured DNA samples were sequenced as paired-end 200-bp reads on the HiSeq2500 platform (Illumina, CA, USA). Functional impacts of the variants were predicted by PolyPhen-2, SIFT, and Mutation Taster prediction software. Variant interpretation was manipulated according to guidelines from the American College of Medical Genetics and Genomics (ACMG) [8]. Sanger sequencing was used to verify genetic mutations which detected by WES. Amplifications were executed with primers targeting the variant loci as follows: forward, 5′-TGAAATGAGACAGTTTAATCCAAAA-3′(chr3:25775467-F-552) and reverse 5′-CAGGAGGCTGAGATACGAGAA-3′(chr3:25775467-R-552) for c.1168C > T and c.1156G > T in NGLY1.

**Fig. 2:**
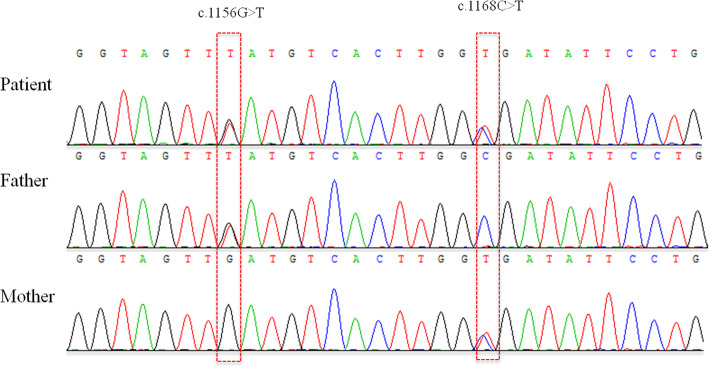
Electropherograms of *NGLY1* exon 8 for the proband and her family. **Patient** The proband carries compound heterozygous mutations (c.1168C > T [p.R390*] and c.1156G > T [p.D386Y]). **Father** Her father carries c.1156G > T. **Mother** Her mother carries c.1168C > T

**Fig. 3:**
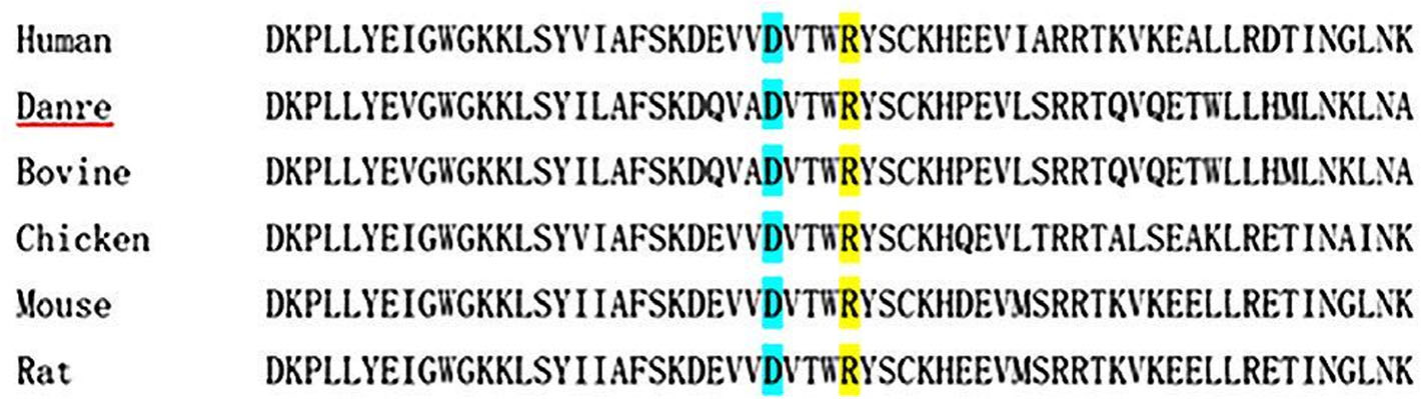
Conservation of the NGLY1 protein sequence among species. The residues mutated in the proband and her family are highlighted

WES identified two compound heterozygous variants in the proband in exon 8 of *NGLY1.* c.1168C > T (p.R390*) had been inherited from her mother, and c.1156G > T (p.D386Y) from her father. The mutations were confirmed by Sanger sequencing (Fig. 2). Since the nonsense mutation c.1168C > T (p.R390*) led to cessation of amino acid translation and was extremely infrequent in GnomAD, ExAC, and 1000 Genomes (PM2). Therefore, c.1168C > T (p.R390*) was classified as “pathogenic” according to the ACMG [8]. Moreover, the missense mutation c.1156G > T (p.D386Y) was absent from the 1000 Genomes Project and extremely infrequent in ExAC and GenomeAD (PM2). Additionally, the results of the prediction software were PolyPhen-2 (score 0.91219), SIFT (score 0.97092), and Mutation Taster (score 0.81033) (PP3). Thus, c.1156G > T (p.D386Y) was considered a variant of uncertain significance (VUS). The two compound heterozygous mutations had not previously been described in association with NGLY1-CDDG, and both were highly conserved among a variety of species (Fig. 3https://genome.ucsc.edu/).

## Discussion and conclusions

NGLY1-CDDG is a rare autosomal recessive hereditary disease, of which a few dozen cases have previously been described [1–7, 9]. The incidence rate of this disease is unknown. Mutation of NGLY1, which encodescytoplasmic protein N-glycanase 1, has been demonstrated as a cause of NGLY1-CDDG. In this report, we present a case involving a Chinese female with NGLY1-CDDG who presented with elevated liver transaminases, developmental delay, epilepsy (subclinical seizures) and constipation. WES identified novel compound heterozygousmutations c.1168C > T/c.1156G > T in exon 8 of *NGLY1* in the patient, which were inherited from her mother and father. Sanger sequencing affirmed these mutations. Termination of the mutation at c.1168C > T leads to cessation of amino acid translation, and the missense mutation c.1156G > T causes an amino acid change (aspartic>tyrosine).

*NGLY1* is located on chromosome 3p24.2, which has 12 exons, and encodes the cytoplasmic protein N-glycanase 1(654-amino acid), which plays an important role in the deglycosylation of cytoplasmic N-linked glycoproteins and glycopeptides [10–12]. In 2012 [5], Need et al. used WES to study unidentified genetic diseases and first reported the relationship between *NGLY1* mutations and congenital glycosylation disorder. To date, many mutations in *NGLY1* have been reported in the Human Gene Mutation Database. The most common pathogenic variant is c.1201A > T, accounting for approximately one-third of pathogenic alleles [4].

N-glycanase 1 functions in the quality control system for newly synthesized glycoproteins in the endoplasmic reticulum, where misfolded glycoproteins are retrotranslocated to the cytosol for degradation, and it also plays a critical role in MHC class I-mediated antigen presentation [13]. Clinical manifestations of NGLY1-deficient patients include abnormal tear production, choreoathetosis, and liver disease, global developmental delay, acquired microcephaly, hypotonia, EEG abnormalities with or without overt seizures, brain imaging abnormalities, peripheral neuropathy, constipation, and a history of intrauterine growth retardation osteopenia, hypocholesterolaemia, difficult swallowing, transient hepatomegaly, anhydrosis, undescended testes, pain insensitivity, low total protein and albumin in the cerebrospinal fluid, and so on [1, 3, 4].

Formal diagnostic criteria of NGLY1-CDDG have not been established. This disorder can be diagnosed if molecular genetic testing finds biallelic pathogenic variants of *NGLY1.* No US Food and Drug Association-approved treatments for NGLY1-CDDG currently exist, but enzyme replacement therapy is being evaluated in the preclinical arena [14]. Moreover, preclinical screens for endo-beta-N-acetylglucosaminidase inhibitors are also underway [15].

Here, we describe a case of NGLY1-CDDG with novel compound heterozygous mutations in NGLY1. Elevated liver transaminases, developmental delay, constipation, and EEG abnormalities with subclinical seizures were found in this patient, which were consistent with previous reports. Both mutations are predicted to abolish NGLY1 protein, and neither has previously been identified. The patient was treated with oral compound glycyrrhizin tablets, and liver transaminase levels were slightly reduced compared to the first presentation.

In summary, we report a case of a Chinese female with NGLY1 CDDG, which a few dozen cases have previously been described. In this case, we provide new gene mutation sites of NGLY1, and this may offer help for the diagnosis of NGLY1-CDDG in the future.

## Data Availability

The raw datasets generated and/or analysed during the current study are not publicly available in order to protect participant confidentiality. The data and materials are available from the corresponding author (ZJ) upon reasonable request.

## Abbreviations

NGLY1-CDDG: NGLY1-related congenital disorder of deglycosylation
WES: Whole-exome sequencing
ERAD: Endoplasmic reticulum-associated degradation
EEG: Electroencephalography
ACMG: American College of Medical Genetics and Genomics
